# Workplace Sitting Breaks Questionnaire (SITBRQ): an assessment of concurrent validity and test-retest reliability

**DOI:** 10.1186/1471-2458-14-1249

**Published:** 2014-12-05

**Authors:** Zeljko Pedisic, Jason A Bennie, Anna F Timperio, David A Crawford, David W Dunstan, Adrian E Bauman, Jo Salmon

**Affiliations:** Faculty of Kinesiology, University of Zagreb, Zagreb, Croatia; Prevention Research Collaboration, Sydney School of Public Health, The University of Sydney, Sydney, Australia; Institute of Sport, Exercise and Active Living (ISEAL), Victoria University, Melbourne, Australia; Centre for Physical Activity and Nutrition Research, School of Exercise and Nutrition Sciences, Deakin University, Melbourne, Australia; The Physical Activity Laboratory, Baker IDI Heart and Diabetes Institute, Melbourne, Australia; School of Population Health, The University of Queensland, Brisbane, Australia; Department of Medicine, Monash University, Melbourne, Australia; Department of Epidemiology and Preventative Medicine, Monash University, Melbourne, Australia; School of Sport Science, Exercise and Health, The University of Western Australia, Perth, Australia

**Keywords:** Breaks in sitting time, Physical activity, Sedentary behaviour, Desk-based employees, Light-intensity physical activity, Reliability, Validity, Accelerometer, Self-report

## Abstract

**Background:**

Breaks in prolonged sitting may have beneficial cardiometabolic and musculoskeletal health outcomes. Desk-based work settings are an important environment to promote and support breaks in sitting time. However, few studies have reported the psychometric properties of self-report measures to assess the frequency and duration of breaks from sitting. This study examined the concurrent validity and test-retest reliability of the Workplace Sitting Breaks Questionnaire (SITBRQ) designed to assess frequency and duration of breaks in sitting within desk-based work settings.

**Methods:**

To assess the concurrent validity, a sample of 147 desk-based employees completed the SITBRQ and wore an Actigraph GT1M accelerometer for seven consecutive days. To establish test-retest reliability, SITBRQ was administered on two separate occasions 7–14 days apart to a separate sample of 96 desk-based employees.

**Results:**

A low relative agreement with accelerometry (Spearman’s r = 0.24 [95% CI 0.07 - 0.40]) was determined for self-reported frequency, but not for the duration of sitting breaks (Spearman’s r = 0.05 [95% CI −0.12 - 0.22]). Adequate reliability was determined for both self-reported frequency (Spearman’s r = 0.71 [95% CI 0.59 - 0.79], Cohen’s kappa = 0.74 [95% CI 0.64 - 0.84]) and duration of sitting breaks (Spearman’s r = 0.59 [95% CI 0.45 - 0.71], Cohen’s kappa = 0.61 [95% CI 0.38 - 0.85]).

**Conclusion:**

SITBRQ may be used for assessment of the frequency of sitting breaks within desk-based work settings with validity and reliability similar to other self-reports in the field of sedentary behaviour research. However, until adequately improved and re-evaluated, it should not be used to collect data about the duration of breaks in sitting time.

**Electronic supplementary material:**

The online version of this article (doi:10.1186/1471-2458-14-1249) contains supplementary material, which is available to authorized users.

## Background

Physical inactivity is one of the leading global public health issues in developed countries [1]. There is well-established epidemiological evidence to suggest that a minimum of 150 minutes of moderate or 75 minutes of vigorous-intensity physical activity a week, or an equivalent combination of both, significantly reduces the risk of a number of chronic diseases [2]. Recently, sedentary behaviour (too much sitting as opposed to too little physical activity) has emerged as a candidate independent risk factor [3]. Several reviews have shown that high volumes of time spent sitting or engaged in sedentary behaviour have been associated with an increased all-cause mortality and chronic disease (e.g., diabetes, cardiovascular disease) and obesity risk [4–6]. In some studies, associations between sedentary behaviour and all-cause mortality and chronic disease risk occurred irrespective of whether an individual meets the public health physical activity recommendations [7–10].

Researchers have recently investigated the potential for health benefits associated with light-intensity physical activity [11, 12]. This type of activity is defined as being between 1.8-2.9 metabolic equivalent units of rest (METs) and is typically non-structured and incidental in nature [13]. Examples of light-intensity physical activity include common habitual free-living activities such as routine occupational (e.g. standing, retail serving and food preparation) or domestic tasks (e.g. ironing, washing up, gardening) [13]. Studies have shown that time spent in light-intensity physical activity is highly inversely correlated (*r* = −0.95) with time spent in sedentary behaviour [14]. Hence, if an individual has low levels of light-intensity physical activity, it is likely that he or she is highly engaged in sedentary behaviours.

While the evidence base is still developing, data from experimental studies [15, 16] and several cross-sectional observational studies [12, 17–19] suggest that higher levels of light-intensity physical activity are associated with a reduced risk of cardiometabolic disease biomarkers and being overweight/obese. Importantly, some of these findings were present even after adjustment for time spent in moderate-to-vigorous physical activity and sedentary behaviour.

These insights have prompted some health agencies and professional societies to provide formal documents outlining some behavioural modification strategies to reduce time spent in sedentary behaviour among adults and children, and suggest strategies generally based around increasing volumes of light-intensity physical activity [20–22]. One of the main strategies is to periodically take short breaks in sitting time within the occupational and home settings [20–22]. It is suggested, for example, that workers periodically take a short break in sitting at least once every 30 minutes, walk to their co-workers instead of telephoning or emailing, stand up during meetings/presentations and install height adjustable sit-stand workstations [22]. Studies have indicated that taking short breaks may assist in reducing detrimental biomechanical consequences of prolonged sitting, such as posture-related musculoskeletal disorders [23] as well as the risk of cardiometabolic disorders and obesity [15, 19, 24, 25].

In addition to spending approximately half their waking hours in this setting [26, 27], employees in computer-centric work environments may be sitting for up to 80% of their working time [28, 29]. It has also been shown that office workers take significantly fewer breaks in sedentary time during working hours compared to non-occupational time [28, 30]. Desk-based and computer-centric work environments may, therefore, be the key behavioural settings to support and promote breaks in sitting time [31].

With the emerging public health interest around the positive health consequences of breaks in sitting time, it is important to develop valid and reliable assessments of this behaviour. Although self-report measures have been the most commonly used method in large-scale epidemiological studies on physical activity and sedentary behavior [32, 33], almost all studies assessing breaks in sedentary behavior have used objective measures, such as accelerometers [19, 24, 34, 35], multi-sensor devices [36], inclinometers [29] and sitting pads [25]. While this may in part be due to reduced cost and increased availability of objective devices in the last decade, few self-report instruments are available and psychometric data are lacking.

Several previous studies have developed self-report instruments that assess occupational physical activity [37–41]. However, these surveys do not ask specifically about short breaks in sitting time. For example, the Occupational Physical Activity Questionnaire (OPAQ) assesses time spent walking at work and doing heavy labor at work [37]. The Occupational Sitting and Physical Activity Questionnaire (OSPAQ) assesses the proportion of occupational time spent sitting, standing, walking, and in heavy labour [38]. The International Physical Activity Questionnaire (IPAQ) asks about the weekly frequency and usual time/day spent in vigorous-intensity physical activity, moderate-intensity physical activity and walking at work [39]. ‘Breaks in occupational sedentary time’ are considered as any interruption in sitting time during work (e.g., going for a bathroom break, walking to a remote printer, standing up for a short stretch, standing while talking to a college), whilst ‘occupational physical activity’ is usually defined in terms of longer periods spent in walking, moderate-intensity or vigorous-intensity physical activity at work (often in ≥10 minute bouts). Hence, occupational physical activity questionnaires may not necessarily capture all interruptions in sitting time. Moreover, the available occupational physical activity questionnaires usually capture total time spent in occupational activities and not the daily frequency of such activities. It is therefore important to develop and validate questionnaires that specifically assess breaks in occupational sedentary time.

Three previous studies have assessed measurement properties of self-reported frequency of breaks in occupational sitting time [42–44]. Clark et al. [42] evaluated an interview-administered item (*“How many breaks from sitting [such as standing up or stretching or taking a short walk] during one hour of sitting would you typically take at work?,”* with the following response scale; *0, 1, 2, 3, 4,* and *≥5*) and observed its weak correlation (r = 0.26) with accelerometer-derived breaks per sedentary hour among office workers. Reliability of the item was not assessed. Furthermore, Lynch et al. [43] developed a past year measure of domain-specific sedentary behaviour (SIT-Q), which includes an item about breaks in occupational sitting time (*“How often did you ‘break up’ the time you spent sitting in job # 1?,”* with the following response scale: *(a) less than hourly; (b) hourly; (c) half hourly; (d) every 10 minutes; (d) every 5 minutes; (f) I did not sit for more than 30 minutes in a day*). The item has shown moderate test-retest reliability (weighted kappa = 0.49), but has not been tested for validity [43]. Furthermore, Wijndaele et al. [44] changed the reference period of SIT-Q from the past year to the last seven days (SIT-Q-7d), and found poor test-retest reliability (ICC = 0.26 and 0.12 for the Dutch and English version respectively) and poor validity tested against activPAL3 monitors (Spearman’s r = 0.06; Dutch version. This shows that more research is needed to further test and improve measurement properties of self-reported frequency of breaks in occupational sitting time.

Further, the total duration of breaks in occupational sitting time is conceptually equal to the total time spent in light- (including standing quietly), moderate- and vigorous-intensity physical activity. We assumed that asking participants about the duration of breaks in sitting time, instead of asking directly about their physical activity, might better capture sporadic and short bouts of light-intensity physical activity. It seems reasonable, however, that longer breaks in sitting time (e.g. 20 minutes or more) would be more easily recalled as occupational activity, rather than as interruptions to sitting. We, therefore, assumed that measuring the duration of sedentary breaks via self-reports may only be feasible among participants within desk-based work settings that typically do not engage in longer bouts of physical activity. No previous studies have assessed the reliability and validity of self-reported duration of breaks in sitting time.

Therefore, the aim of this study was to develop and evaluate concurrent validity and test-retest reliability of the *Workplace Sitting Breaks Questionnaire* (SITBRQ; Additional file 1), which includes items on frequency and duration of breaks in occupational sitting time.

## Methods

Concurrent validity and reliability of the SITBRQ were tested in two separate samples (Validity Sample; Reliability Sample) between February and April 2009. Written informed consent was obtained from the organisations and employees involved. The study protocols were approved by the Deakin University Ethics Committee (EC 207–2009).

### Participants and procedures

#### Validity sample

To test validity, a convenience sample of 143 employees who worked a minimum of four days a week was recruited. This was a sub-sample of a larger study described elsewhere [28]. In brief, a large organisation located in metropolitan Melbourne, Australia participated in the study. The organisation had various offices and retail outlets in metropolitan Melbourne, and an internal staff email was sent to employees to promote the study. This was followed up by trained research staff visiting each workplace to administer study materials to those employees wishing to participate. The participants completed a survey and were asked to then wear an accelerometer for the next seven consecutive days.

#### Reliability sample

The present study used a sub-set of participants from a larger population survey conducted in metropolitan Melbourne, Australia [45]. A random sample of 316 workplaces from Melbourne, Australia were approached and 55 organizations agreed to participate (response rate = 17.4%). Contacts within each recruited workplace were asked to distribute materials to staff whose typical working tasks involved being seated at a desk or workstation (such as office administration, data entry and any other desk-bound occupations). A total of 1467 surveys were distributed with 722 returned (response rate = 49.2%). Participants who completed the questionnaire were asked whether they would agree to participate in the test-retest reliability study by completing an abbreviated version of the survey that included SITBRQ, on a second occasion 7–14 days after completion of the initial survey. Similar time frame was used when assessing test-retest reliability of self-reported measures of sedentary behaviour in most previous studies [32]. The between-subject variability in the timeframe of the second survey was inevitable, due to the study design. Questionnaires were sent directly to all participants who agreed to take part in the second survey (n = 96). Completed surveys were returned to the researchers via a reply-paid envelope.

#### Measures

##### *The Workplace Sitting Breaks Questionnaire*(SITBRQ)

The SITBRQ was developed to assess the frequency and duration of breaks in sitting time within the context of desk-based work settings. When constructing self-report instruments to measure complex behaviours, it is recognised that the use of relevant cues and examples of the behaviours of interest is essential for the design of effective assessment tools [46]. As breaks in sitting time is a relatively new concept, the idea was explored in one-on-one interviews with a convenience sample of 33 employees who typically sit for working tasks, in order to determine the most effective terminology. Most described the term ‘short physical activity breaks’ as the most succinct way to describe breaks in sitting time during work hours (unpublished data). The term ‘short physical activity breaks’ was, therefore, used and defined as ‘any interruption in sitting time during work hours’. To further aid understanding, we provided a ‘preamble’ to the question on reporting frequency and duration of short physical activity breaks at work, which gave examples of this behaviour.

Participants were asked to report how many breaks in sitting time they would take during a work hour on a typical work day (categorical response options ranged from 0 to ≥6) and the total time per day typically spent in short physical activity breaks at work (six categorical response options were provided: <5, 5–9, 10–19, 20–29, 30–59, and ≥60 minutes). The SITBRQ item about the frequency of breaks in sitting time was developed based on the single-item questionnaire by Clark et al. [42]. The wording of the SITBRQ question is almost identical to its source. The corresponding response scale includes 7 instead of 6 options that were originally included in Clark et al. [42], because we assumed that an additional category may reduce the number of participants potentially affected by the measurement ceiling effect. The wording of the SITBRQ item about the duration of breaks in sitting time was not based on any questionnaires, as no such items were previously published.

#### Accelerometry

A uniaxial accelerometer (Actigraph GT1M, Pensacola, FL, USA) was used to establish the concurrent validity of SITBRQ. The Actigraph accelerometers have shown acceptable validity against doubly-labelled water [47] and have often been used as a concurrent measure to validate physical activity and sedentary behaviours questionnaires [48]. Participants were asked to wear the accelerometer on their right hip during all waking hours for 7 days (5 work and 2 non-work days) and to record accelerometer on/off times, as well as work start and finish times in an event diary [28]. In the present study we only used accelerometer data collected during work hours. Only participants who wore accelerometers for five or more hours of their working time on at least three days were included in the analysis (n = 135). Accelerometer data were summarized using SAS 9.1 (SAS Institute, Cary, NC, USA). Previously commonly used cut points were used to classify the accelerometer data, with sedentary time defined as <100 counts/minute (cpm) [32], low-intensity as ≥100-1951 cpm and moderate to vigorous-intensity physical activity as ≥1952 cpm [49]. As shown in Thorp, et al. [28], the employees’ working hours were mostly spent sedentary on 77% or 6.6 hours/day). Most of the remaining time was comprised of light-intensity physical activity (on 20% or 1.7 hours/day), with minimal moderate-to-vigorous physical activity (on 2% or 0.2 hours/day) recorded. Light-intensity physical activity and moderate-to-vigorous physical activity were combined to classify time spent in all physical activities as being ≥100 cpm. All the minutes of accelerometry with ≥100 cpm were summed to form the total duration of breaks in sedentary time. Every change between <100 cpm and ≥100 cpm was counted as a break in sitting time. No minimum duration for break or a minimum duration of sedentary epochs before a break was set. The same approach to determine breaks in sitting time was used in previous studies [24].

#### Socio-demographic profile

Participants reported their age, sex, level of education, employment status and main occupation. These were collapsed as follows: age as 18–29, 30–39, 40–49, 50–59, and ≥ 60 years; employment status as full time, and part-time; educational attainment as <12 years, ≥12 years, trade or technical, and university or tertiary qualification; and occupational domain as managers/administrators, professionals, associate professional, tradespersons, advanced clerical, intermediate clerical, and other (coded according to the Australian Standard Classification of Occupation (ASCO) coding system) [50].

#### Data analysis

Concurrent validity was assessed by Spearman’s rank correlations. There are no specifically intended accelerometer cut-points for assessment of breaks in sitting time (i.e., changes from sitting to standing posture) and the number of minutes classified as sedentary seems to be inversely related to the level of sedentary behaviour threshold (i.e., higher sedentary behaviour thresholds are associated with lower number minutes classified as sedentary) [51]. Accelerometer-based measures can, therefore, only be considered as relative estimates of breaks in sitting time. Taking this into account, only the relative agreement between SITBRQ and accelerometer-based measures (as expressed by Spearman’s rank correlations) was tested, whilst the absolute agreement was not hypothesised. Furthermore, Spearman’s rank correlations were calculated to evaluate the relative agreement between test and retest. Quadratic weighted Cohen’s kappa coefficients of agreement, percentage of responses correctly classified, percentage of responses in same or adjacent category, and percentage of highly misclassified responses were calculated to evaluate the absolute agreement between test and retest. The measure of relative agreement, therefore, represents how proportionate were participants’ responses on two administrations of the questionnaire. The measures of absolute agreement illustrate the proportion of identical and non-identical responses on the test and retest. To allow for generalization, 95% confidence intervals were provided for all statistics. The validity and reliability were assessed by Spearman’s rank correlations, because SITBRQ data is ordinal-scaled. The choice of the method is in accordance with previous studies [32, 42, 44]. The data analyses were conducted using STATISTICA, version 10 (StatSoft, Inc., Tulsa, OK, USA) and IBM SPSS Statistics 21 (SPSS Inc. an IBM Company, Chicago, IL, USA).

## Results

Table 1 shows socio-demographic characteristics and self-reported frequency and duration of sitting breaks from the respective validity and reliability samples. Both samples comprised greater proportions of women (63%) than men (37%). Across both samples, most participants were aged less than 60 years (96% in both samples), were in full-time employment (95% in the validity and 86% in the reliability sample) and had a university or tertiary qualification (62% in the validity and 69% in the reliability sample). Approximately one in two participants reported being managers or professionals (45% in both samples). Furthermore, over 80% of participants in both samples reported having between one and three sitting breaks per working hour. Few participants reported a total duration of sitting breaks of ≥60 minutes (3.1% in the validity and 5.2% in the reliability sample). The average number of sedentary breaks per hour and the total duration of breaks (in minutes), as assessed by accelerometers in the validity sample, was 5.67 ± 1.58 and 124 ± 42 (mean ± standard deviation), respectively.

**Table 1:** **Sample characteristics, and frequency and duration of sitting breaks**

	Reliability sample (%)	Validity sample (%)
	***(n = 96)***	***(n = 143)***
**Sex**		
Men	37.5	37.4
Women	62.5	62.6
**Age (years)**		
18-29	26.0	33.5
30-39	27.4	33.8
40-49	25.6	19.7
50-59	16.9	9.2
60 and over	4.2	2.1
**Education**		
Some high school	8.3	7.1
Year 12 or equivalent	16.7	25.4
Trade or technical	6.3	9.3
University/tertiary qualification	68.8	61.8
**Occupation**		
Managers/administrators	23.7	25.2
Professionals	20.9	19.7
Associate professional	15.3	16.2
Trades persons	3.5	0.0
Advanced clerical	17.5	15.9
Intermediate clerical	19.1	23.0
**Employment Status**		
Full-time	86.1	94.6
Part-time	13.9	4.8
**Frequency of sitting breaks** ^*^		
0	0.0	1.5
1	35.4	26.5
2	25.0	31.8
3	19.8	25.8
4	6.3	6.8
5	7.3	7.6
≥ 6	6.3	0.0
**Duration of sitting breaks (minutes)** ^*^		
< 5	14.6	20.9
5 - 9	13.5	15.5
10 - 19	37.5	21.7
20 - 29	12.5	15.5
30 - 59	16.7	23.3
≥ 60	5.2	3.1

^*^Assessed by the Workplace Sitting Breaks Questionnaire (SITBRQ).

Spearman’s rank correlation showed low relative agreement between the SITBRQ and accelerometry in estimating the frequency of breaks (Spearman’s r = 0.24 [95% CI 0.07 - 0.40]) (Table 2). No agreement was found between the SITBRQ and accelerometer-based estimates of the total duration of breaks (Spearman’s r = 0.05 [95% CI −0.12 - 0.22]).

**Table 2:** **Concurrent validity of the Workplace Sitting Breaks Questionnaire (SITBRQ) against accelerometer-based measures** ^*^

Questionnaire item	Spearman’s rho (95% CI) ^†^
**Frequency of breaks (breaks/hour)**	0.24 (0.07 - 0.40)
**Total duration of breaks (minutes/day)**	0.05 (−0.12 - 0.22)

*Accelerometer-based measures (total time in ≥100 cpm) were categorized to reproduce response scales of questionnaire items.

^†^Spearman’s rank correlation between SITBRQ and accelerometer-based measures and its 95% confidence interval.

The Spearman’s rank correlation coefficients showed somewhat higher test-retest reliability of SITBRQ in estimating frequency of breaks than in estimating the total duration of breaks (Spearman’s r; 0.71 [95% CI 0.59 - 0.79] vs. 0.59 [95% CI 0.45 - 0.71]) (Table 3). According to Landis and Koch [52] the agreement between categorical responses in test and retest was substantial (Cohen’s kappa = 0.74 [95% CI 0.64 - 0.84]) and moderate (Cohen’s kappa = 0.61 [95% CI 0.38 - 0.85]) for the frequency of breaks and the total duration of breaks, respectively. According to the reported frequency of breaks, the majority of participants (85% [95% CI 78% - 93%]) were classified in the same or adjacent category between survey administrations. For the total duration of breaks, 79% (95% CI 71% - 87%) participants selected the same or adjacent response category in the test and retest.

**Table 3:** **Test-retest reliability** ^*^ **of the Workplace Sitting Breaks Questionnaire (SITBRQ)**

Questionnaire item	Spearman’s rho (95% CI) ^†^	Cohen’s kappa (95% CI) ^‡^	% correctly classified (95% CI) ^§^	% in same or adjacent category (95% CI) ^||^	% highly misclassified (95% CI) ^¶^
**Frequency of breaks (breaks/hour)**	0.71 (0.59 - 0.79)	0.74 (0.64 - 0.84)	51.0 (41.0 - 61.0)	85.4 (78.4 - 92.5)	14.6 (7.5 - 21.6)
**Total duration of breaks (minutes/day)**	0.59 (0.45 - 0.71)	0.61 (0.38 - 0.85)	46.9 (36.9 - 56.9)	79.2 (71.0 - 87.3)	20.8 (12.7 - 29)

^*^Test and retest surveys were conducted a maximum of 14 days apart.

^†^Spearman’s rank correlation between test and retest and its 95% confidence interval.

^‡^Quadratic weighted Cohen’s kappa coefficient of agreement between test and retest and its 95% confidence interval.

^§^Percent of participants classified in same categories in test and retest its 95% confidence interval.

^||^Percent of participants classified in same or adjacent categories in test and retest its 95% confidence interval.

^¶^Percent of participants classified in distant categories in test and retest (two or more categories apart) its 95% confidence interval.

## Discussion

The SITBRQ was designed as a brief self-report instrument to assess the frequency and duration of breaks in sitting among employees who commonly sit for working tasks. The current study demonstrated low concurrent validity of the SITBRQ for the assessment of frequency of breaks in sitting time and no correlation with accelerometer-based estimates of duration of the breaks. The questionnaire showed good reliability for the assessment of frequency and duration of breaks in sitting time.

The correlation between self-reported and accelerometer-based frequency of breaks in sitting time determined in our sample (Spearman’s r = 0.24) was consistent with that reported by Clark et al. [42] (Spearman’s r = 0.26) and higher than the one found by Wijndaele et al. [44] (Spearman’s r = 0.06). A review article by Helmerhorst et al. [53] showed that the Spearman’s correlation coefficient between questionnaire-based and accelerometer-derived time in sedentary behaviour is usually around 0.23. Hence, the relative agreement between the self-reported and accelerometer-based frequency of breaks in sitting time determined in our study is similar to other self-reports in the field of sedentary behaviour research. The low concurrent validity does not necessarily imply that this item is not valid. The concurrent validity was tested by assessing a bidirectional correlation between two concurrent instruments (the SITBRQ and accelerometers). The magnitude of the correlation, therefore, depended on the validity of both instruments, and may have been low because accelerometer cut-points are not specifically developed to capture short interruptions in sitting time. It seems that the SITBRQ may have potential to rank individuals based on their frequency of breaks in sitting time. However, we acknowledge that this assumption needs to be further tested by assessing its criterion validity against a ‘gold standard’ measure of frequency of breaks in sitting time (e.g. using inclinometers worn on the thigh).

Our results showed that the SITBRQ does not provide valid estimates of the total duration of breaks in sitting time. Light-intensity physical activities that are most often performed in sitting breaks are typically unstructured and sporadic, which makes them difficult to recall [46]. The measure requires participants to sum the time spent in all breaks during a usual working day. From a cognitive perspective, this might be a more demanding task than recalling the usual frequency of breaks per hour [46]. A subsequent analysis revealed that almost all misclassified participants (i.e., those who were not classified in same categories based on accelerometry and SITBRQ) had higher duration of sedentary breaks assessed by accelerometer when compared to the self-reported estimate (data not shown). The highest category on the response scale (60 minutes or more) was selected by very few participants, while the responses were evenly distributed across all other categories. Therefore, it seems unlikely that the relative agreement between SITBRQ and accelerometer-based estimates was compromised by the restricted range of the scale. Nevertheless, it might be that asking an open-ended question or providing a response scale with a greater number of categories would increase the relative agreement with accelerometer-based estimates. This type of measure could be tested in future validation studies. Until adequately improved and re-evaluated, this item should not be used to collect data about the duration of breaks in sitting time.

Participants’ responses showed good relative and absolute agreement across two survey occasions as expressed by Spearman’s correlations and Cohen’s kappa coefficients, respectively. This demonstrates the ability of SITBRQ to reliably rank and classify participants according to their self-reported frequency and duration of breaks in sitting time. Reliability of both SITBRQ items was similar as for most other physical activity and sedentary behaviour questionnaires [53] and can therefore be considered satisfactory. The SITBRQ has shown somewhat higher reliability in assessing frequency of breaks in sitting time than SIT-Q [44]. This difference may, however, be explained by the shorter interval between two administrations of the questionnaire used in our study when compared to Lynch et al. [44] (7–14 days vs. one month). It is possible that the second administration of the questionnaire in our study was under a greater influence by carryover effects due to memory. Furthermore, our participants assessed the frequency of breaks somewhat more reliably than the duration of breaks. This is in accordance with the study of McCormack et al. [54], who found higher reliability for the self-reported frequency than for the self-reported duration of incidental physical activities.

A key limitation of this study was the lack of a true ‘gold standard’ to objectively measure frequency and breaks in sitting time, namely, accelerometers have several limitations as sedentary behaviour measures. The accelerometer cut-point used here to assess breaks in sitting time (≥100 cpm) is the commonly used threshold for differentiation between sedentary behaviour and physical activity [32]. However, this cut-point is not specifically intended for detection of changes from sitting to standing posture (i.e., breaks in sitting time). Researchers suggest that behaviours such as fidgeting legs whilst seated may result in accelerometer readings above 100 cpm [17, 24]. Therefore, the objective and self-report instruments used in the present study may have not captured the same behaviours, with accelerometry potentially overestimating frequency and duration of breaks by detecting movements undertaken while seated. To avoid this problem, future validation studies should therefore consider using other objective instruments that more specifically measure the behaviour of interest, such as inclinometers that are able to detect sit-to-stand transitions and non-sitting/lying time. Furthermore, recording accelerometer data in 1-minute epochs increased the likelihood not to capture short sit-stand transitions, which made the discrepancies between SITBRQ and accelerometer-based estimates even more probable. In addition, SITBRQ had been administered before the accelerometer measures were taken. Although SITBRQ asks about breaks in sitting time on a typical day, it may be that this has caused a mismatch between the timeframe of self-reported and accelerometer-based estimates, and further lowered their agreement. Furthermore, the concurrent validity was assessed in a convenience sample, which may have reduced the generalizability of our findings.

Strengths of the study include a relatively large sample size, and a critical evaluation of a specifically designed instrument that captures both frequency and duration of breaks in sitting time within the context of work settings. This study was the first to evaluate the concurrent validity and reliability of the self-reported duration of breaks in sitting time.

## Conclusion

The SITBRQ item for the assessment of frequency of breaks in sitting time using showed concurrent validity similar to other self-reports in the field of sedentary behaviour research and satisfactory reliability, indicating its potential for utilization in future studies. Nevertheless, additional efforts may be needed to improve its validity. Furthermore, despite satisfactory reliability, the item related to self-reported duration of breaks in sitting time did not demonstrate acceptable concurrent validity. Therefore, this item in its current form should not be used to collect data about the duration of breaks in sitting time. Additional research is needed to further refine and validate self-report measures of breaks in sitting time among employees within desk-based work 9pt?>settings. Future studies might benefit from establishing the validity of SITBRQ against measures that are more suitable for assessing breaks in sitting time than accelerometers.

## Electronic supplementary material

Additional file 1: **Workplace Sitting Breaks Questionnaire (SITBRQ).** (DOCX 15 KB)
