# Supplementary material for: Workplace Sitting Breaks Questionnaire (SITBRQ): an assessment of concurrent validity and test-retest reliability
Source: BMC Public Health. 2014 Dec 5;14:1249. doi: 10.1186/1471-2458-14-1249 (PMC4289328; doi:10.1186/1471-2458-14-1249)
Supplement: Supplementary file 1 — Additional file 1: Workplace Sitting Breaks Questionnaire (SITBRQ). (DOCX 15 KB) [file 12889_2014_7351_MOESM1_ESM.docx]

**Workplace Sitting Breaks Questionnaire (SITBRQ)**

**Short Physcial Activity Breaks = any interruption to sitting time at work**

*One area we wish to explore is whether you take breaks from your working tasks during work hours. During a typical working day there may be opportunities to take short physical activity breaks from your working tasks. This is any interruption to your sitting time at work. For example, taking time away from your desk/workstation to move around perhaps to get a drink, have a bathroom break or even continuing a work task while standing.*

How many breaks from sitting (such as standing up, or stretching or taking a short walk) during one hour of sitting would you typically take at work?

(*please circle the correct answer to the following questions*)

| 1. During a typical work day how many **breaks from sitting** (such as standing up, or stretching or taking a short walk) during **Uone hour** of sitting would you take **Uat workU**? | 6  or more | 5 | 4 | 3 | 2 | 1 | 0 |
| --- | --- | --- | --- | --- | --- | --- | --- |
| 1. What is the **Utotal time**U you spend in **short physical activity breaks** during a **typical day** at work? | 60 minutes or more | 30-59 min | 20-29 min | 10-19 min | 5-9 min | Less than  5 min | N/A |
